# Plant‐derived protein bodies as delivery vehicles for recombinant proteins into mammalian cells

**DOI:** 10.1002/bit.27273

**Published:** 2020-01-30

**Authors:** Jennifer Schwestka, Marc Tschofen, Stefan Vogt, Sylvain Marcel, Johannes Grillari, Marianne Raith, Ines Swoboda, Eva Stoger

**Affiliations:** ^1^ Department of Applied Genetics and Cell Biology University of Natural Resources and Life Sciences Vienna Austria; ^2^ Department of Biotechnology, Institute of Molecular Biotechnology University of Natural Resources and Life Sciences Vienna Austria; ^3^ iBio CDMO Bryan Texas; ^4^ Christian Doppler Laboratory for Biotechnology of Skin Aging University of Natural Resources and Life Sciences Vienna Austria; ^5^ Ludwig Boltzmann Institute for Experimental and Clinical Traumatology Vienna Austria; ^6^ Biotechnology Section, FH Campus Wien University of Applied Sciences Campus Vienna Biocenter Vienna Austria

**Keywords:** bioencapsulation, molecular farming, plant‐made pharmaceuticals, protein bodies, recombinant proteins

## Abstract

The encapsulation of biopharmaceuticals into micro‐ or nanoparticles is a strategy frequently used to prevent degradation or to achieve the slow release of therapeutics and vaccines. Protein bodies (PBs), which occur naturally as storage organelles in seeds, can be used as such carrier vehicles. The fusion of the N‐terminal sequence of the maize storage protein, γ‐zein, to other proteins is sufficient to induce the formation of PBs, which can be used to bioencapsulate recombinant proteins directly in the plant production host. In addition, the immunostimulatory effects of zein have been reported, which are advantageous for vaccine delivery. However, little is known about the interaction between zein PBs and mammalian cells. To better understand this interaction, fluorescent PBs, resulting from the fusion of the N‐terminal portion of zein to a green fluorescent protein, was produced in *Nicotiana benthamiana* leaves, recovered by a filtration‐based downstream procedure, and used to investigate their internalization efficiency into mammalian cells. We show that fluorescent PBs were efficiently internalized into intestinal epithelial cells and antigen‐presenting cells (APCs) at a higher rate than polystyrene beads of comparable size. Furthermore, we observed that PBs stimulated cytokine secretion by epithelial cells, a characteristic that may confer vaccine adjuvant activities through the recruitment of APCs. Taken together, these results support the use of zein fusion proteins in developing novel approaches for drug delivery based on controlled protein packaging into plant PBs.

## Graphical abstract

In this study, the use of plant‐made protein bodies (PBs) for protein delivery into mammalian cells was explored. Fluorescent PBs produced in *N. benthamiana* leaves were recovered by a filtration‐based downstream procedure and incubated with human colon epithelial and macrophage‐like cells. PBs were internalized into mammalian cells at a higher rate than polystyrene beads of comparable size and stimulated cytokine secretion by epithelial cells. The findings support the development of zein‐based PBs as a drug delivery vehicle.

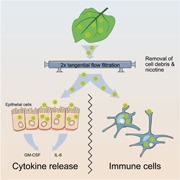

## 1 INTRODUCTION

Oral administration of pharmaceuticals is often the desired drug delivery route for reasons such as safety, patient compliance, and socioeconomic advantages (De Smet, Allais, & Cuvelier, 2014; Sastry, Nyshadham, & Fix, 2000). Oral vaccines, for instance, have the additional benefit of being able to elicit not only immunoglobulin G‐mediated serum immunity but also immunoglobulin A (IgA)‐mediated mucosal immunity, thus providing an advantage since many pathogens enter the host through mucosal surfaces (Breedveld & van Egmond, 2019). However, a major challenge for oral therapeutics is the need for them to withstand the harsh conditions of the gastric system, such as low pH and digestive enzymes. To ensure that the active components remain intact upon arrival at their effector site, they need to be fortified to prevent degradation. One way to achieve such robustness is by encapsulating therapeutics into micro‐ or nanoparticles.

Zein, a prolamin‐type storage protein from maize seeds, is extensively used for encapsulation purposes because it is biocompatible and biodegradable (Luo & Wang, 2014) and was generally recognized as safe for oral use by the US Food and Drug Administration in 1985 (Zhang et al., 2015). There are several ways in which zein can be used for encapsulation purposes. Most studies have used in vitro methods such as phase separation, spray drying, supercritical antisolvent technique, emulsification/solvent evaporation, or chemical crosslinking techniques (Zhang et al., 2016). Most in vitro encapsulation studies using zein have focused on the incorporation of poorly water‐soluble, nonproteinaceous compounds like curcumin (Patel, Hu, Tiwari, & Velikov, 2010), aceclofenac (Karthikeyan, Vijayalakshmi, & Korrapati, 2014), quercetin (Penalva, González‐Navarro, Gamazo, Esparza, & Irache, 2017), or alpha‐tocopherol (Luo, Zhang, Whent, Yu, & Wang, 2011), but these methods have also been used to encapsulate lysozyme (Zhong & Jin, 2009) and the antioxidant proteins catalase and superoxide dismutase (S. Lee, Alwahab, & Moazzam, 2013; S. Lee, Kim, & Park, 2016).

Alternatively, zein‐containing protein storage organelles, so‐called zein protein bodies (PBs), found in maize endosperm cells (Lending & Larkins, 1989), may offer natural bioencapsulation strategies for recombinant oral pharmaceuticals. This assumption has been substantiated by experiments with rice seeds showing that the sequestration of recombinant proteins in endogenous storage organelles containing rice prolamins confers protection from digestive proteolysis after oral administration in an animal model (Nochi et al., 2007). A faster and more versatile method for encapsulating proteins into the protective environment of zein micro/nanocarriers is to create a fusion protein in which the protein of interest is fused to a partial sequence of zein. Expression of such fusion protein results in in vivo bioencapsulation in various production hosts, within newly induced storage organelles. Amongst the various classes of zeins: α (19 and 22 kDa), β (15 kDa), γ (16, 27, and 50 kDa), δ (10 kDa; Woo, Hu, Larkins, & Jung, 2001)—the 27 kDa γ‐zein was identified as the key element that induces the formation of endogenous as well as recombinant PBs. Furthermore, it was discovered that the N‐terminal 93 amino acids of 27 kDa γ‐zein (abbreviated gz93 from here on) are sufficient to produce PBs in other plants, and even in heterologous expression systems such as fungal, insect, and mammalian cells (Llop‐Tous et al., 2010; Torrent et al., 2009). Various proteins with different properties in terms of molecular mass and function, including growth factors (Torrent et al., 2009), viral vaccine candidate proteins (Hofbauer et al., 2016; Mbewana, Mortimer, Pêra, Hitzeroth, & Rybicki, 2015; Whitehead et al., 2014), and enzymes (Llop‐Tous, Ortiz, Torrent, & Ludevid, 2011), have been successfully incorporated into newly induced PBs in plants like *Nicotiana benthamiana* when fused to gz93. *N. benthamiana* is frequently used for the production of biopharmaceuticals because it is well suited for the transient expression of recombinant proteins, and this method offers advantages over other expression systems in terms of speed, safety, scalability, and reduced upstream production costs. However, the cost savings in the upstream process are sometimes offset by industrial downstream processes for the purification of biopharmaceuticals, which are often quite laborious and may account for approximately 70–80% of the total manufacturing costs regardless of the expression host (Schillberg, Raven, Spiegel, Rasche, & Buntru, 2019). In the case of orally delivered plant‐made products, the complexity of the downstream process could be reduced and plant tissues could be administered after minimal processing, allowing to take maximum benefit of the competitive upstream production costs offered by plants.

Previously, it was reported that zein PBs can have an adjuvant effect when administered by injection. For example, the fusion of a therapeutic HPV vaccine candidate to the Zera® peptide, a self‐assembly domain very similar to gz93, enhanced the immune responses in mice (Whitehead et al., 2014). Similarly, when we fused hemagglutinin‐5 (H5) to gz93, the resulting PBs were able to elicit a strong immune response that was on par with soluble H5 plus Freund's complete adjuvant, while soluble H5 without adjuvant failed to induce an immune response (Hofbauer et al., 2016). Particulate formulations of antigens generally show this immunostimulatory effect and one possible explanation is that upon internalization of a single particle, many copies of the antigen enter the cell, whereas a much higher dose must be administered to achieve comparable local concentrations surrounding the cell (Colino et al., 2009; Snapper, 2018). Alternatively, the enhanced immune response may also be due to superior antigen display and stability or other immunostimulatory signals (Smith, Simon, & Baker, 2013). In addition, gz93 harbors eight repeats of a proline‐rich domain (VHLPPP)_8_ that closely resembles the sweet arrow peptide (VRLPPP)_3_, which is known for having cell‐penetrating properties (Sánchez‐Navarro, Teixidó, & Giralt, 2017).

In the present study, we focus on the potential of PBs for oral application. We explore a downstream procedure based on two consecutive tangential flow filtrations (TFFs) as a means to enrich the zein PBs from larger amounts of leaf tissue, and we investigate the internalization efficiency of zein PBs into cells of the mucosal lining by comparing the uptake of fluorescent gz93 PBs and polystyrene beads of comparable size. We demonstrate efficient PB internalization into intestinal epithelial cells as well as antigen‐presenting cells (APCs). Finally, we analyze whether the epithelial cells secrete cytokines, which are known to recruit APCs.

## 2 MATERIALS AND METHODS

### 2.1 Molecular cloning

The coding sequences of gz93‐enhanced green fluorescent protein (eGFP) and gz93‐mTagBFP2 were designed in silico and synthesized by GeneCust, Europe. The sequences were then cloned into the pTRA vector, a derivative of pPAM (GenBank AY027531), by restriction cloning using *Smi*I and *Xba*I cut sites. The translated sequence starts with the N‐terminus of 27 kDa γ‐zein (GenBank accession number: AF371261) including its native signal peptide and the first 93 amino acids of the mature protein (hence gz93), followed by a short flexible (GGGGS)_2_ linker, which finally connects to the eGFP or the monomeric blue fluorescent protein (mTagBFP2; Subach, Cranfill, Davidson, & Verkhusha, 2011). gz93‐eGFP is expressed under control of a 35S promoter with a duplicated transcriptional enhancer and a 35S terminator, both originating from *Cauliflower mosaic virus*. In addition, the transcribed region contains a 5′‐untranslated region from *Tobacco etch virus*, which confers the increased stability of the messenger RNA. Two matrix attachment regions of tobacco Rb7 (Halweg, Thompson, & Spiker, 2005) flank the promoter and terminator up‐ and downstream, respectively, to suppress transgene silencing.

### 2.2 Plant material and agroinfiltration

*N. benthamiana* plants were cultivated in the soil in a growth chamber with a 16 hr photoperiod at 70% relative humidity and day/night temperatures of 26°C and 16°C, respectively. The gz93‐eGFP and gz93‐mTagBFP2 plasmids were transferred into chemically competent *Agrobacterium tumefaciens* GV3101‐pMP90RK. Cultures of this *Agrobacterium* strain were inoculated from glycerol cryo‐stocks and cultivated in YEB medium containing 25 mg/L kanamycin, 25 mg/L rifampicin, and 50 mg/L carbenicillin. Cultures were incubated at 28°C while shaking at 200 rpm. Before infiltration, the cultures were pelleted and washed twice with infiltration medium (10 mM MES pH 5.6, 10 mM MgCl_2_, 100 µM acetosyringone) and adjusted to OD_600_ 0.2 with infiltration medium. The infiltration of *N. benthamiana* leaves was performed manually with 1 ml syringes. Leaves were harvested 8 days postinfiltration (dpi) for the production of PBs for uptake assays, while smaller samples for size determination were harvested at 4 and 12 dpi as well.

### 2.3 PB size determination

The diameter of gz93‐eGFP PBs was determined at 4, 8, and 12 dpi by analyzing the maximum projected z‐stacks of confocal laser scanning microscopy (CLSM) pictures. For each sample, a 5 × 5 mm section was excised from the agroinfiltrated leaves of *N. benthamiana* and mounted on a glass slide with tap water as the immersion medium. The samples were observed under a Leica SP5 Confocal Laser Scanning Microscope using a ×63 water immersion objective (NA 1.20). The Argon laser power was set to 16% and the 488 nm laser line was set to 2% output for the excitation of eGFP. Forty‐eight pictures along the *z*‐axes were recorded at a resolution of 1,024 × 1,024 pixels for each picture with a step size of 1.1 µm (bidirectional scanning at 400 Hz, 2x line averaging). Maximum projections of z‐stacks were exported from Leica Software and analyzed using Adobe Photoshop. In total, 832, 986, and 821 individual PBs from at least three samples per time point were measured for 4, 8, and 12 dpi, respectively.

### 2.4 Processing of plant material

*N. benthamiana* leaf material expressing gz93‐eGFP or gz93‐mTagBFP2 was harvested at 8 dpi and stored at −20°C until processing. Leaf material, 200 g, was homogenized in a Waring‐type blender with the addition of 800 ml phosphate‐buffered saline (PBS) extraction buffer (137 mM NaCl, 2.7 mM KCl, 10 mM Na_2_HPO_4_, 1.8 mM KH_2_PO_4_, pH 7.4) supplemented with 2% Triton X‐100. The extract was further homogenized with a disperser (IKA ULTRA‐TURRAX® S 25 N‐10 G) and then repeatedly pelleted by centrifugation at 15,000 rcf for 30 min at 4°C. The supernatants were discarded, and the pellets were washed twice with PBS extraction buffer including 2% Triton X‐100 and twice with PBS lacking Triton X‐100. The resulting suspension was then filtered through a 180 µm nylon mesh filter utilizing a vacuum‐assisted bottle‐top filter holder. Small amounts of antifoam Y‐30 were added when necessary. This was then subjected to the first TFF using a nylon filter cloth with a 10 µm cut‐off. Since TFF systems with this pore rating were not available, we built a prototype TFF filter holder that can be equipped with any cloth or membrane. This filter holder provided a surface area of 96 cm^2^ and was operated by a peristaltic pump. gz93‐eGFP PBs passed through the 10 µm filter and the permeate was washed and concentrated using a second TFF with a 0.65 µm cut‐off (C02‐E65U‐07‐N; Spectrum Labs). Once some of the permeate had passed the first filter, both systems could be operated simultaneously. The concentrated retentate was subjected to low‐speed density centrifugation over a cushion of 40% CsCl (1.4225 g/cm^3^) at 4,800 rcf for 30 min at 20°C. The top layer was collected and washed twice with five sample volumes PBS, to remove CsCl, by pelleting at 21,000 rcf for 5 min at 20°C.

### 2.5 Flow cytometry of PBs

Processed samples of gz93‐eGFP PBs were measured in a V‐bottom 96‐well plate and data were collected for 10,000 events using a flow cytometer (CytoFlex S; Beckman Coulter). eGFP signal was excited at 488 nm and emission was measured at 525 nm. Forward, side scatter, and eGFP gain was set to 40, 24, and 50, respectively. To show the reproducibility of the method, three independent measurements, each including five replicates, were performed. Flow cytometry data were analyzed with CytExpert 2.3 (Beckman Coulter).

### 2.6 Determination of nicotine content

Nicotine extraction was performed as described (Moghbel, Ryu, & Steadman, 2015). PBs derived from 50 mg of leaves (FW) were extracted for 2 hr in a 1‐ml extraction solution (40% aqueous methanol containing 0.1% 1 N hydrochloric acid). The supernatant was collected and the pellet was re‐extracted twice. The supernatants were combined and evaporated to dryness (Savant Speed Vac SC‐110 with cooling unit RVT‐100; Savant instruments, Holbrook). For comparison, 50 mg of leaves of *N. benthamiana* were ground and extracted according to the same protocol. A nicotine standard (N0267; Merck, Germany) was used for quantification. For high performance liquid chromatography‐electrospray ionization‐ tandem mass spectrometry (HPLC‐ESI‐MS/MS) measurements, the sample was dissolved in 12 μl of 80 mM ammonium formate buffer (pH 3.0) and 5 μl was loaded on a BioBasic C18 column (BioBasic 18, 150 × 0.32 mm, 5 µm; Thermo Fisher Scientific, Waltham, MA) using a Dionex UltiMate 3000 system directly linked to a QTOF instrument (maXis 4G ETD; Bruker). A gradient from 99.0% to 6.2% of solvent A and 1.0–93.8% of solvent B (solvent A: 80 mM ammonium formate buffer at pH 3.0, B: 80% acetonitrile and 20% A) was applied over a 10 min interval at a flow rate of 6 μl/min. The mass spectrometer was equipped with the standard ESI source and measurements were performed in positive ion, DDA mode (= switching to MSMS mode for eluting peaks). MS scans were recorded (range, 100–1,500 *m*/*z*) and the four highest peaks were selected for fragmentation. Instrument calibration was performed using an ESI calibration mixture (Agilent).

### 2.7 Mammalian cell culture

Human colonic epithelial cells (HCEC‐1CT, CkHT‐039‐0229; Evercyte GmbH, Vienna) were routinely grown at 37°C under a humidified atmosphere of 7% CO_2_ in DMEM:199 (4:1/Biochrome, Germany) supplemented with 2% cosmic calf serum (HyClone, Logan, UT), EGF (25 ng/ml), hydrocortisone (1 g/ml), insulin (10 g/ml), transferrin (2 g/ml), and sodium selenite (5 nM; all from Sigma‐Aldrich, St. Louis, MO). Differentiation of cells toward colonic epithelial cells (described by Roig et al., 2010) was induced by culturing cells for 48 hr in DMEM:199 (4:1) supplemented with 0.1% cosmic calf serum, EGF (1.25 ng/ml), hydrocortisone (1 µg/ml), insulin (10 µg/ml), transferrin (2 µg/ml), sodium selenite (5 nM), and GSK‐2 inhibitor IX (5 µm; Merck).

U937 cells (ATCC CRL 1593) were cultivated in Roswell Park Memorial Institute 1640 media (Biochrom, Germany) containing 10% heat‐inactivated fetal calf serum and 4 mM l‐glutamine (Sigma‐Aldrich). Differentiation of cells toward macrophage‐like cells was induced by culturing 7 × 10^5^ cells/ml in medium containing 100 nM phorbol 12‐myristate 13‐acetate for 24 hr. The medium was changed to routine medium and cells were cultivated for further 48 hr until the cells attached to the surface showing the development of a dendritic‐like morphology.

### 2.8 PB uptake and flow cytometry of HCEC cells

For uptake studies, the medium was supplemented with 100 units/ml of penicillin, and 100 μg/ml of streptomycin (Sigma‐Aldrich) and 2 × 10^4^ cells/cm^2^ were seeded and differentiated for 48 hr until confluence was reached. On the basis of the results from the quantification of PBs using a flow cytometer, cells were incubated with 150 gz93‐eGFP PBs/cell at 37°C (*n* = 3) for 2, 6, 12, 18, and 24 hr. Before cell detachment using 0.1%/0.02% Trypsin/EDTA for 5 min, the cells were washed thoroughly with PBS to remove the remaining particles. The uptake of fluorescent particles into the cells was analyzed in a flow cytometer (CytoFlex S; Beckman Coulter). Yellow–green‐labeled 1‐µm polystyrene microspheres (F13081; Thermo Fisher Scientific) were used for comparison. As a negative control, cells were kept for 6 hr at 4°C to prevent active particle uptake. The negative control was carried out with 150 gz93‐eGFP PBs or polystyrene microspheres (PS beads) per cell, respectively, and the signal obtained was subsequently subtracted from the fluorescent signal obtained from cells incubated at 37°C.

The supernatant of cells was collected after 24 hr of incubation either with or without gz93‐eGFP PBs or PS beads, centrifuged for 15 min with 500 rcf at 4°C, and stored at −80°C until analysis of the cytokine content. Cytokines (interleukin‐6 [IL‐6], granulocyte‐macrophage colony‐stimulating factor [GM‐CSF]) secreted into the media by HCEC‐1CT cells, were measured using the MILLIPLEX® MAP Human Cytokine/Chemokine panel (HCYTOMAG‐60K; Merck Millipore, Burlington, MA). As a positive control, cells were incubated for 24 hr with 200 ng/ml tumor necrosis factor‐α (Sigma‐Aldrich) to induce cytokine secretion.

### 2.9 Microscopy of mammalian cells

HCEC cells were seeded in eight‐well microslides (Cat #80826; ibidi, Gräfelfing, Germany) and differentiated after a cell number of 3 × 10^4^ cells/cm^2^ was reached. After incubation with gz93‐eGFP PBs for 4 hr, cells were washed three times with PBS. To visualize the cell nuclei, cells were stained for 5 min with 2 µg/ml Hoechst 33342 (H1399; Thermo Fisher Scientific). In addition, cells were incubated for 5 min at 37°C with 5 µg/ml of the lipophilic cell membrane dye FM4‐64 (T13320; Thermo Fisher Scientific). The cellular uptake of PBs into HCEC‐1CT was confirmed by CLSM using a Leica TCS SP8 with a ×63 water immersion objective (NA 1.20; Leica Microsystems, Wetzlar, Germany; lasers: diode 405 nm, white light laser 488 nm, 565 nm; detectors: HyD 432–472 nm, PMT 503–515 nm). Z‐stacks were generated with a step size of 0.7 µm.

U937 cells were incubated for 2 hr with gz93‐eGFP and gz93‐mTagBFP2 PBs at 37°C. Lysosomes of cells were loaded by pulsing cells with 0.1 mg/ml Alexa Fluor 647 Dextran for 4.5 hr, and then cells were studied for 2 hr. Cellular uptake of PBs into U937 cells was confirmed by CLSM as described above.

## 3 RESULTS

### 3.1 Gz93‐eGFP PBs produced in *N. benthamiana* leaves are enriched by a filtration‐based process

**Figure 1:**
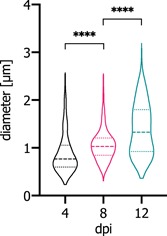
Size determination of gz93‐eGFP PBs. Samples of *Nicotiana benthamiana* were analyzed by CLSM at 4, 8, and 12 dpi. The diameter of PBs increases significantly over time as determined by one‐way ANOVA with post hoc Tukey's test (****p < .0001). ANOVA, analysis of variance; CLSM, confocal laser scanning microscope; dpi, days post infiltration; eGFP, enhanced green fluorescent protein; PBs, protein bodies [Color figure can be viewed at wileyonlinelibrary.com]

For the production of fluorescent recombinant PBs, eGFP was genetically fused to the C‐terminus of gz93 connected by a flexible (GGGGS)_2_ linker. The resulting construct (gz93‐eGFP) was transferred to leaves of *N. benthamiana* by agroinfiltration and the formation of spherical PBs was confirmed by CLSM (Figure S1A). The size distribution of PBs was analyzed at 4, 8, and 12 dpi, and median diameters of 0.77 (*SD* ± 0.43), 1.03 (*SD* ± 0.34), and 1.33 (*SD* ± 0.55) µm were determined, respectively (Figure 1), indicating that gz93‐eGFP particles keep increasing in size. We chose to harvest PBs at 8 dpi with a median diameter of 1.03 µm for cellular uptake studies.

**Figure 2:**
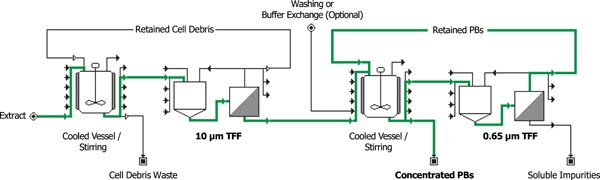
A scalable process for the enrichment of zein PBs, based on two consecutive tangential flow filtrations. At the first step, cell debris is retained by a 10‐µm nylon filter while PBs are able to pass and the second step concentrates the PBs while allowing soluble contaminants to permeate. In this process flow chart, the path of PBs is highlighted in green. PBs, protein bodies [Color figure can be viewed at wileyonlinelibrary.com]

To obtain sufficient amounts of PBs, we developed a new downstream procedure for the enrichment of zein PBs that is based on a combination of filtration steps (Figure 2) and therefore more easily scalable than previously described processes based on ultracentrifugation (Hofbauer et al., 2016; Whitehead et al., 2014). Our procedure comprises initial washing steps with buffer containing Triton X‐100 to solubilize membranes and to remove soluble host proteins and other compounds from the insoluble fraction. This was followed by coarse straining through a 180 µm mesh and two subsequent TFFs with pore sizes of 10 and 0.65 µm, respectively. The first TFF removes large cell debris while gz93‐eGFP PBs pass through the filter. The second TFF step was carried out to remove additional soluble host proteins and particles that are smaller than gz93‐eGFP PBs. Through this procedure, it was possible to reduce the sample volume and concentrate it by a factor of 100. As a result, much more of the sample could be subjected to centrifugation over a cushion of 40% CsCl (1.4225 g/cm^3^) that allows separating particles with a higher density than gz93‐eGFP PBs (e.g., starch granules). In addition, this step is performed at 4,800 rcf, and this enables more of the sample to be processed compared with procedures where centrifugation is done at ultrahigh speeds (>50,000 rcf).

The resulting preparations of gz93‐eGFP PBs were evaluated by flow cytometry. This method allowed us to identify two populations of particles with distinct fluorescence properties (Figure S2). In agreement with visual inspection by confocal microscopy, we concluded that the population of fluorescent particles represents gz93‐eGFP PBs while the rest is probably cell debris. The mean concentration of fluorescent particles (*n* = 3) was 3.18E+06 events/µl (*SD* ± 13.2%) corresponding to 5.12E+07 gz93‐eGFP PBs/g fresh weight of leaves.

The nicotine levels of *N. benthamiana* leaves and of the gz93‐eGFP PB preparation were determined using HPLC‐ESI‐MS/MS (Table S1). The nicotine content in *N. benthamiana* leaves was around 47,500 ng/g, whereas the residual nicotine content in a PB sample derived from 1 g of leaves was 3.89 ng (*SD* ± 0.2), demonstrating that during the downstream procedure, nicotine was depleted by a factor of 1.22E+04. The residual amount of nicotine is comparable with the nicotine content found in some vegetables. For example, the levels of nicotine in the edible parts of tomato and eggplant are 3–7 ng/g (Moldoveanu, Scott, & Lawson, 2016), and according to Andersson, Wennström, and Gry (2003), the average nicotine exposure from consumption of vegetables is approximately 1,000 ng/day.

### 3.2 Gz93‐eGFP PBs are endocytosed by human colon epithelial cells and stimulate cytokine release

**Figure 3:**
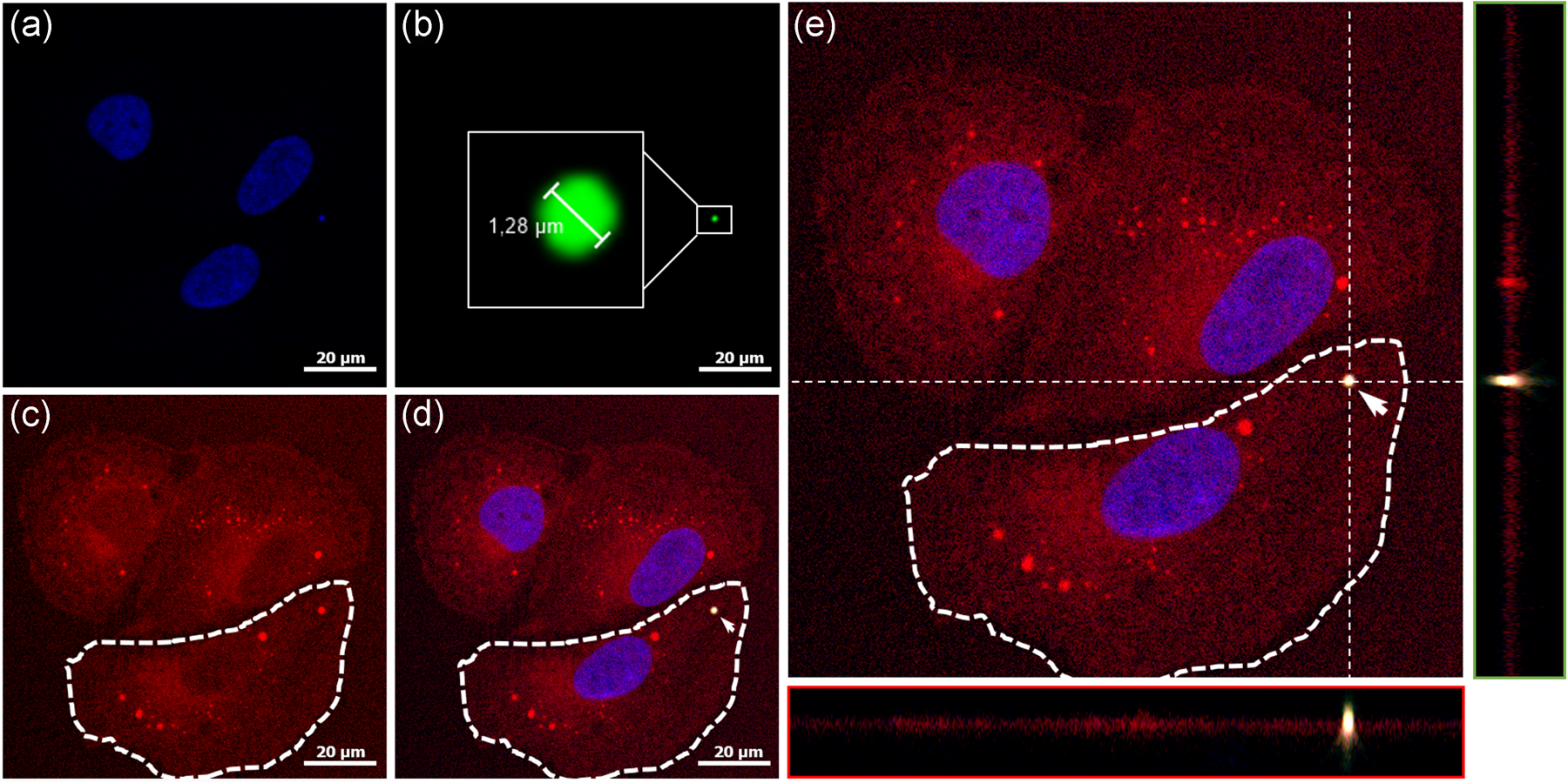
HCEC‐1CT cells have internalized gz93‐eGFP PBs after 4 hr of incubation. The cell nucleus was stained with Hoechst 33342, displayed in blue (a), gz93‐eGFP PBs emit green fluorescence (b), and the cell membrane and intracellular structures were stained in red with FM4‐64 (c). When all channels are merged (d) an orange signal results from the overlay of the green and red fluorescence that indicates the internalization of gz93‐eGFP PB into HCEC‐1CT cells. The cell was imaged in 32 sections (with a step size of 0.7 µm), and the cell is shown in the *xy*‐axis at *z* = 11. *yz*‐ (e, green panel) and *xz*‐projections (e, red panel) clearly confirm the internalization of the analyzed PB (arrow). The bar represents 20 µm. eGFP, enhanced green fluorescent protein; PBs, protein bodies [Color figure can be viewed at wileyonlinelibrary.com]

To demonstrate the endocytosis of zein PBs into cells of the human intestinal barrier, we used the human colon epithelial cell line HCEC‐1CT (immortalized by hTERT and CDK4), which maintains expression of cell‐type‐specific markers and functions of colon epithelial cells (Roig et al., 2010). The uptake of gz93‐eGFP PBs into HCEC‐1CT cells was demonstrated by CLSM and quantified by flow cytometry. CLSM images showed that cells are able to take up gz93‐eGFP PBs within 4 hr of incubation (Figure 3a–d). The cellular internalization of a gz93‐eGFP PB was confirmed by providing optical sections (*xy*‐) with *xz*‐ and *yz*‐projections (shown in Figure 3e), which allowed a clear differentiation between extracellular and internalized PBs. Furthermore, the internalization is proven by the overlay of the green signal, originating from the gz93‐eGFP PB, and the red signal emitted by FM4‐64 reported to stain endocytic membranes (Hansen, Rasmussen, Niels‐Christiansen, & Danielsen, 2009).

**Figure 4:**
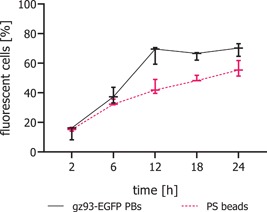
Uptake of gz93‐eGFP PBs by HCEC‐1CT cells in comparison to fluorescent polystyrene beads. The relative number of fluorescent HCEC‐1CT cells was determined by flow cytometry at the indicated time points. Values are shown as medians with their 95% confidence interval. eGFP, enhanced green fluorescent protein; PBs, protein bodies [Color figure can be viewed at wileyonlinelibrary.com]

A second experiment was carried out to quantitatively assess the uptake of PBs by flow cytometry and to compare the uptake efficiencies of PBs and PS beads. On the basis of the quantification of fluorescent events per µl, 150 gz93‐eGFP PBs or PS beads per cell were added to in vitro cultures of HCEC‐1CT cells and incubated for 2, 6, 12, 18, and 24 hr. Endocytosis of gz93‐eGFP PBs occurred faster than that of PS beads, as indicated by a sharper increasing curve for the PBs, reaching a plateau after 12 hr (Figure 4). Mean values after 12 hr reached 66.5% (*SD* ± 6.2) and 43.5% (*SD* ± 4.9) for gz93‐eGFP PBs and PS beads, respectively. The difference of 22.9% (*SD* ±  4.6) was significant in Student's *t* test (*p* < .01). Also, after exposure for 18 and 24 hr, the overall number of fluorescent cells incubated with PS beads remained below the levels obtained with gz93‐eGFP PBs (*t* test; *p* < .05).

**Figure 5:**
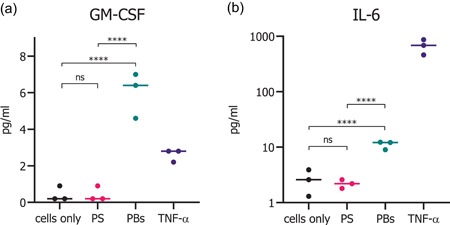
Cytokine secretion of HCEC‐1CT cells after 24 hr of incubation with gz93‐eGFP PBs and PS beads. Cells incubated with gz93‐eGFP PBs showed a significant increase in GM‐CSF and IL‐6 secretion compared with cells treated with polystyrene beads. Untreated cells (cells only) were used as a negative control and TNF‐α treated cells as a positive control. Values for three independent experiments are shown and the median values are indicated. *^ns^p* > .05, ****p ≤ .0001. eGFP, enhanced green fluorescent protein; GM‐CSF, granulocyte‐macrophage colony‐stimulating factor; IL‐6, interleukin‐6; PBs, protein bodies; TNF‐α, tumor necrosis factor‐α [Color figure can be viewed at wileyonlinelibrary.com]

Having confirmed that human colon epithelial cells are able to endocytose gz93‐eGFP PBs, we investigated whether endocytosis might lead to the secretion of cytokines that can activate the immune system. Amongst others, the cytokine GM‐CSF is known to have an activating effect on APCs, like macrophages and dendritic cells (Hamilton, 2002). We thus collected the culture medium supernatants from the uptake assays (*n* = 3) and subjected them to Luminex assays. The secretion of GM‐CSF was only elevated upon administration of 150 gz93‐eGFP PBs per cell but not after treatment with the same amount of PS beads (Figure 5a). IL‐6 levels were also significantly increased upon incubation with gz93‐eGFP PBs as compared with the same dose of PS beads (Figure 5b).

### 3.3 PBs are taken up by immune cells

**Figure 6:**
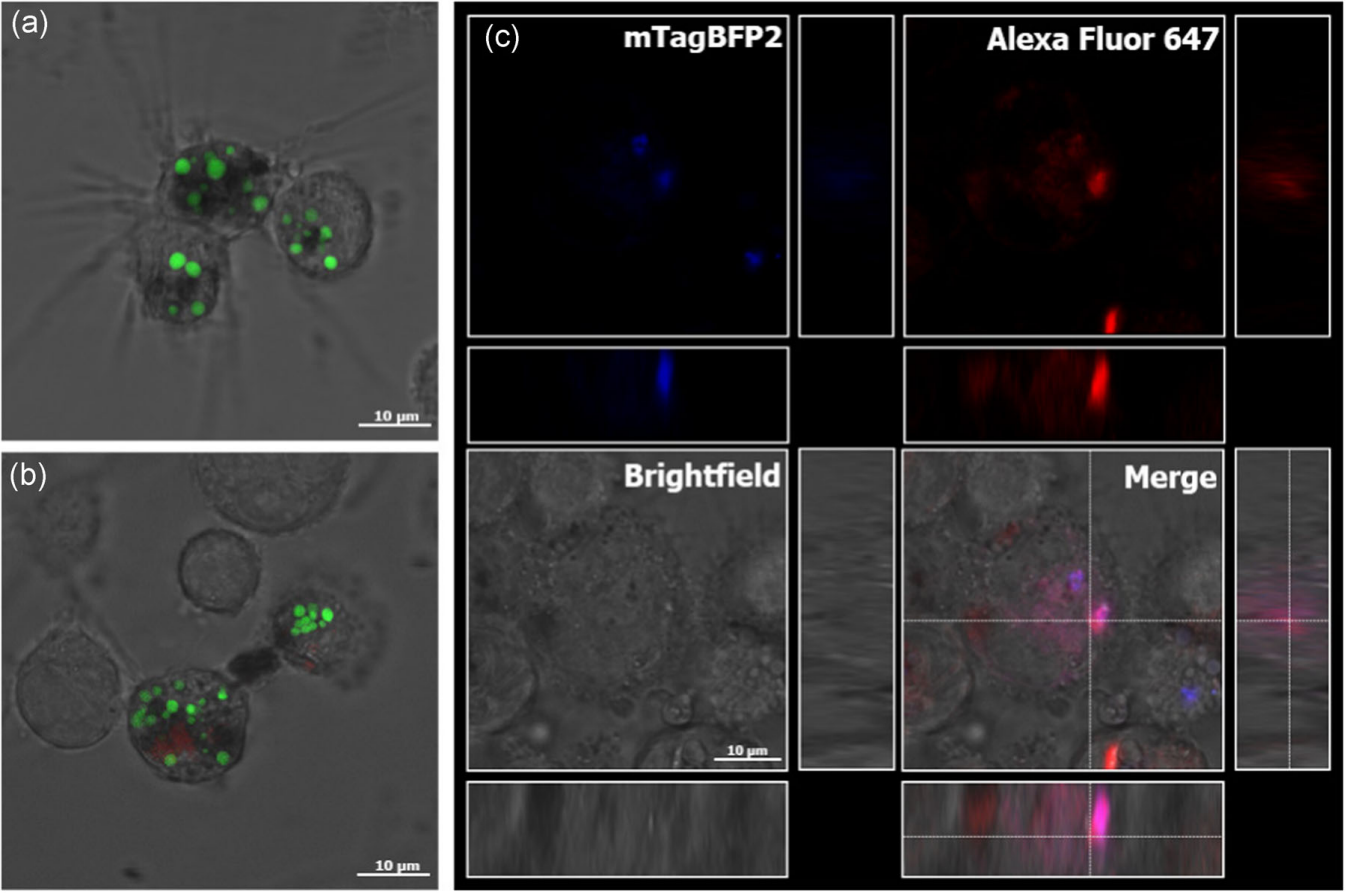
The uptake of gz93‐eGFP and gz93‐mTagBFP2 PBs into U937 cells was confirmed by CLSM. Upon PMA treatment, cells attached to the vessel surface and exhibited morphological changes such as the growth of dendrite‐like structures (a). Endosomal compartments were loaded and visualized with Alexa Fluor 647 Dextran (red, b). Colocalization of blue fluorescent gz93‐mTagBFP2 PBs with endosomal compartments (red) results in a purple signal (c, merge). The bar represents 10 µm. CLSM, confocal laser scanning microscope; eGFP, enhanced green fluorescent protein; PBs, protein bodies; PMA, phorbol 12‐myristat‐13‐acetat [Color figure can be viewed at wileyonlinelibrary.com]

We then investigated the uptake of fluorescent PBs into immune cells using the human myelomonocytic cell line U937. Cells were first matured and differentiated as could be seen by decreased cell proliferation and attachment to the surface. After 2 hr, most of the cells had taken up multiple gz93‐eGFP PBs (Figure 6a,b). To obtain information about the localization of the internalized particles, we visualized the endosomal compartments of those cells with Dextran Alexa Fluor 647, which is known to be internalized in late endosomes after endocytosis (Johnson, Ostrowski, Jaumouillé, & Grinstein, 2016). Since eGFP fluorescence is not stable in the acidic environment of late endosomes, we also used for this experiment PBs containing mTagBFP2, a blue fluorescent protein variant with a pKa of 2.7 ± 0.2 (Subach et al., 2011). In the gz93‐mTagBFP2 PBs, GFP was replaced with mTagBFP2, but otherwise, they were produced and recovered in the same manner as described for the gz93‐eGFP PBs and had a similar appearance and size (Figure S1B). We were able to observe the colocalization of gz93‐mTagBFP2 PBs in compartments stained with Dextran Alexa Fluor 647 (Figure 6c). It is, therefore, likely that the PBs are transported to the late endosomes, where usually antigen processing takes place.

## 4 DISCUSSION

The induction of mucosal immunity by subunit vaccines is a promising prophylactic strategy, which is especially effective against enteric infections (Ghaffari Marandi, Zolfaghari, Kazemi, Motamedi, & Amani, 2019; Qadri, Svennerholm, Faruque, & Sack, 2005; Shojaei Jeshvaghani et al., 2019). However, protein‐based oral vaccines require the protection of the antigen to ensure sufficient stability and transport to the intestine and the underlying immune system (Davitt & Lavelle, 2015; Kour, Rath, Sharma, & Goyal, 2018). Encapsulation of antigenic payload in nanoparticles made of chitosan or synthetic polymers like poly(dl‐lactic‐co‐glycolic acid) (PLGA) provides such protection and has been used as a successful strategy to induce sIgA antibodies upon oral administration (Edelman et al., 1993; Fattal, Pecquet, Couvreur, & Andremont, 2002).

In this study, we focused on using zein PBs as alternative oral drug delivery vehicles since they combine several beneficial properties: Zein PBs have been shown to be recalcitrant against digestion by various proteases (S. H. Lee & Hamaker, 2006), have an adjuvant effect (Hofbauer et al., 2016; Whitehead et al., 2014), and they can mediate the sustained release of in vitro encapsulated small molecule drugs and even DNA (Acevedo et al., 2018; Farris, Brown, Ramer‐Tait, & Pannier, 2017; Regier, Taylor, Borcyk, Yang, & Pannier, 2012; Zhang et al., 2015). In addition, the encapsulation in zein PBs can be achieved directly in the plant production host as an integral part of the upstream process.

For the induction of a mucosal immune response, uptake of an antigen at the intestinal surface is crucial. However, little is known about the ability of zein PBs to interact with mammalian cells. We have, therefore, investigated the uptake of fluorescent zein PBs into human intestinal epithelial and APCs. We could show that the PBs are internalized into intestinal epithelial cells at a higher rate than synthetic polystyrene beads. After 12 hr of incubation, the proportion of cells that had taken up fluorescent PBs reached 66.5% (*SD* ± 6.2). In contrast, after 12 hr of incubation with PS beads, only 43.5% (±4.9) of cells had internalized PS particles, and the number of fluorescent cells reached only 56.1% (±5.3) after 24 hr. This enhanced uptake efficiency of PBs might be due to the amphipathic proline‐rich repeat found in the N‐terminal sequence of γ‐zein, which favors interaction with membranes (Kogan et al., 2004) and is assumed to have cell‐penetrating effects that could promote cellular uptake (Fernández‐Carneado, Kogan, Castel, & Giralt, 2004).

In addition to the uptake of fluorescent PBs, we also showed an immunostimulatory effect on the cells, resulting in an increased secretion of chemoattractant molecules such as GM‐CSF. GM‐CSF is involved in the differentiation of granulocytes and macrophages and in the activation and proliferation of neutrophils, macrophages, and dendritic cells (Hamilton, 2002). With respect to mucosal immunization, the presence of GM‐CSF was shown to increase antigen‐specific antibody production (Okada et al., 1997). GM‐CSF also promotes IL‐6 secretion (Evans, Shultz, Dranoff, Fuller, & Kamdar, 1998), and accordingly IL‐6 levels were also elevated when cells were subjected to PBs. Both chemokines play a pivotal role in the initiation of a humoral response to antigenic proteins (Tada, Hidaka, Kiyono, Kunisawa, & Aramaki, 2018), and IL‐6 has been explored as a molecular adjuvant for mucosal vaccines (Rath et al., 2013; Su et al., 2008; Thompson & Staats, 2011). The observed cytokine release indicates the PB formulation's potential to enhance immunity and to exert an adjuvant effect, which is in agreement with the findings of Whitehead et al. (2014) and Hofbauer et al. (2016).

In addition to antigen uptake via intestinal epithelial cells, dendritic cells can capture antigens directly from the intestinal lumen by extending dendrites through the epithelium (Rescigno et al., 2001). Since GM‐CSF is known to recruit dendritic cells to the subepithelial layer (Egea, Hirata, & Kagnoff, 2010), it is feasible that its secretion would lead to an increased number of dendrites reaching through tight junctions. Therefore, we investigated the uptake of PBs into APCs using the monocytic model cell line U937 (Altaf & Revell, 2013). APCs have the ability to internalize particles of various sizes with high efficiency. CLSM images confirmed the uptake of multiple PBs per cell. To obtain information about the subcellular localization of the internalized particles, we visualized the endosomal compartments with Dextran Alexa Fluor 647 and we used PBs containing a blue fluorescent protein that remains stable in the acidic environment found in late endosomes and lysosomes. Indeed, several particles colocalized with fluorescently labeled endosomal organelles, indicating that PBs might be processed within the endolysosomal system. From there, peptides can be loaded onto major histocompatibility complex class II molecules, which is the prerequisite for a successful immune response (Blum, Wearsch, & Cresswell, 2013; Roche & Furuta, 2015).

Particulate vaccine strategies have been reported to be effective at lower antigen doses compared to soluble formulations (De Smet et al., 2014), but oral vaccines generally require a higher dose of antigen to induce an immune response when compared to traditional parenteral immunizations (Pavot, Rochereau, Genin, Verrier, & Paul, 2012). This presents a challenge in the development of oral vaccine applications, and the corresponding production platforms need to be highly scalable. Even though plant‐based production systems are very flexible with respect to upstream production, the downstream processing procedure often includes rate‐limiting bottlenecks. For example, in most previous reports, the isolation of PBs from leaf material involved a density gradient ultracentrifugation step (Hofbauer et al., 2016; Joseph et al., 2012; van Zyl, Meyers, & Rybicki, 2017). In the present study, the PBs were recovered by a newly established enrichment process based on several low‐speed centrifugations and TFF steps, which can be easily adapted to kg amounts of leaf material without the need to invest in expensive large equipment for continuous ultracentrifugation. The removal of nicotine during the process was demonstrated, and the residual amount of nicotine in the sample was comparable to the nicotine content found in widely consumed vegetables (Moldoveanu et al., 2016). We have also demonstrated that fluorescent zein PBs can be analyzed and quantified by flow cytometry. It is likely that the procedure can also be adapted for nonfluorescent particles by using antigen‐specific antibodies with fluorescent labels, thereby providing a general procedure for quality control of particulate formulations. It is important to note that oral vaccine formulations do not require the extensive purification and sterile conditions necessary for injected formulations, and downstream processing procedures reported for plant‐made oral vaccine candidates range from simple homogenization or minimal processing of plant material to partial purification (Chan & Daniell, 2015; Loza‐Rubio et al., 2012; Merlin, Pezzotti, & Avesani, 2017; Pniewski et al., 2018). The presence of plant‐derived contaminants such as cell wall debris or starch particles, which cannot be completely removed by filtration and density centrifugation steps, are therefore unlikely to constitute a regulatory problem. On the contrary, biocompatible plant constituents, such as starch microparticles, have even been studied as vaccine adjuvants (Rydell & Sjöholm, 2004; Stertman, Lundgren, & Sjöholm, 2006).

In conclusion, we have shown that zein PBs produced in *N. benthamiana* leaves can be recovered by a newly developed filtration‐based downstream procedure and we observed their efficient internalization into cultured cells of the intestinal epithelium as well as APCs. These results support the further development of novel approaches for in planta protein drug encapsulation and delivery, including the design of functionalized multicomponent PBs with defined structures and uptake kinetics for the bioencapsulation of pharmaceutical proteins.

## CONFLICT OF INTERESTS

The authors declare that there are no conflict of interests.

## Supporting information

Supporting information
