# Supplementary material for: Plant‐derived protein bodies as delivery vehicles for recombinant proteins into mammalian cells
Source: Biotechnol Bioeng. 2020 Jan 30;117(4):1037–47. doi: 10.1002/bit.27273 (PMC7079162; doi:10.1002/bit.27273)
Supplement: Supplementary file 1 — Supporting information [file BIT-117-1037-s001.docx]

**
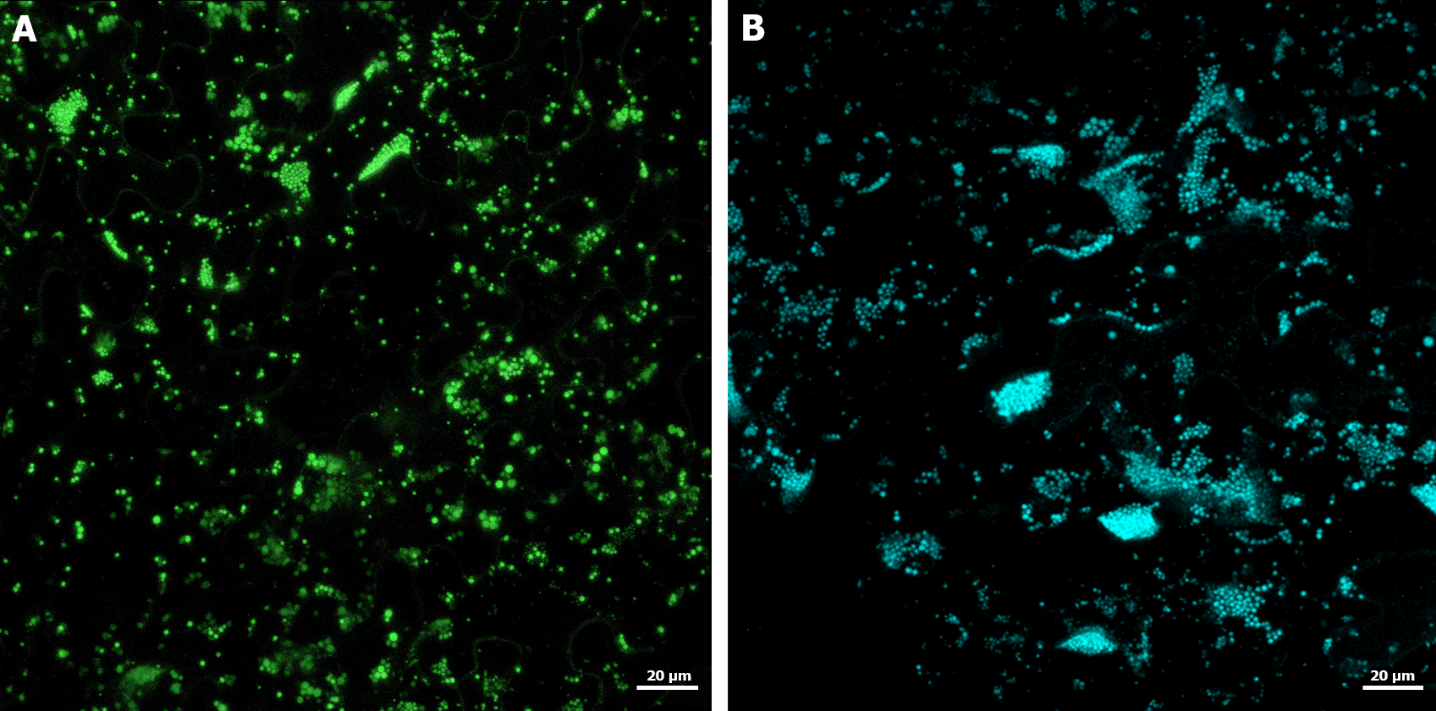
**

**Supplemental figure S1.** Leaf cells of *Nicotiana benthamiana* are expressing (**A)** gz93-eGFP or **(B)** gz93-mTagBFP2 PBs at 8 dpi. Z-stacks of 48 CSLM pictures were displayed as maximum projections and the median diameter of 986 individual PBs was determined to be 1.03 µm (SD±0.34). Scale bars represent 25 µm or 50 µm, respectively.


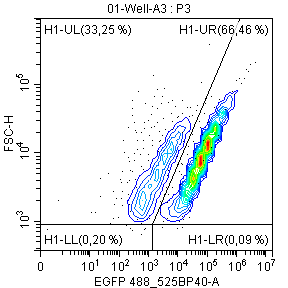


**Supplemental figure S2**. Example contour plot of a flow cytometry measurement of gz93-eGFP PBs. The fluorescence intensity is plotted against the forward scatter (height, FSC-H) and the gz93-eGFP PBs are gated in the upper-right quadrant (H1-UR). The relative amount in this example is 65.6% (SD±0.6) fluorescent events in H1-UR versus 34.4% (SD±0.6) non-fluorescent events in H1-UL.

**Supplemental Table 1**. Nicotine content of *N. benthamiana* leaves and PB samples.

|  | **Nicotine content [ng/g FW]**  **(**±SD) | | | **Nicotine content [µg/g FW]**  **(**±SD) | | |
| --- | --- | --- | --- | --- | --- | --- |
|  |  |  |  |  |  |  |
| gz93-eGFP PBs | 3,89 | ± | 0,20 | 3,89E-03 | ± | 0,00020353 |
| leaves | 47501 | ± | 20258 | 47,50 | ± | 20,258 |
